# The Lived Experiences of Spousal Bereavement and Adjustment Among Older Chinese Immigrants in Calgary

**DOI:** 10.1007/s10823-023-09477-3

**Published:** 2023-04-01

**Authors:** Qianyun Wang, Christine A. Walsh, Hongmei Tong

**Affiliations:** 1 Faculty of Social Work, University of Calgary, University Dr NW, Calgary, AB T2N 1N4 Canada; 2 Department of Social Welfare, The University of California, Los Angeles, California United States; 3 Faculty of Health and Community Studies, MacEwan University, Edmonton, Canada

**Keywords:** Spousal bereavement, Lived experiences, Immigration, Chinese immigrant, Older adult

## Abstract

Spousal bereavement poses considerable challenges to adults in late life. Some populations, such as older immigrants, may experience heightened negative outcomes as a consequence of spousal bereavement, due to migratory stress and social isolation. Spousal bereavement is culturally embedded as it is related to cultural beliefs and attitudes concerning death and family relationships. However, studies on spousal bereavement or widowhood among older immigrants are extremely limited. This study aims to fill the gap by exploring, via a phenomenological approach, the lived experiences of widowed older Chinese immigrants in Calgary and responding to the question: What are the lived experiences of widowed Chinese older immigrants in coping with their spousal bereavement? With the data drawn from 12 in-depth qualitative interviews, findings were categorized into individual, family, community and societal levels. Study participants experienced long-lasting grief that was private and impacted by their culture and immigration status. Although family and ethno-cultural communities provided various types of supports during participants’ widowhood, they did not directly assist them in coping with spousal loss. Most participants did not access social services for bereavement support, more often relying on cultural rituals and faith practices. Findings suggest the need for culturally appropriate bereavement supports and family/community involvement for older immigrant adults who have experienced spousal loss.

## Introduction

Grief caused by the loss of a spouse, termed spousal bereavement, is one of the most stressful events that older adults will experience (Holmes & Rahe, 1967). Spousal bereavement is a common, yet culturally embedded phenomenon; that is, it is highly related to cultural beliefs and attitudes concerning death (Jin & Chrisatakis, 2009; Saito, 2013). Specifically, scholars have identified that Chinese people are influenced by traditional Chinese culture, such as Taoism, Confucianism, and Buddhism, in relation to death and dying (Hsu et al., 2009; Lee 2009). Confucianism, for example, views an individual as deeply embedded in the family, which differs from the Western ideology of individualism. Therefore, Chinese older adults prefer to remain closely involved with their family in old age and including when they are dying (Lee 2009). Further, dying at home has a special cultural meaning for Chinese people, it is deemed as a way of continuing bonds with their ancestors (Hsu et al., 2009). Also, beliefs derived from Taoism and Buddhism lead Chinese people to accept death as a natural phase of life according to “Tian Yi”, or the will of Heaven (Hsu et al., 2009; Lee 2009).

Further, for some older populations, for example, Chinese older immigrants, spousal bereavement and widowhood may be further complicated by factors such as social isolation, economic hardship and migratory stress (Lai & Chau, 2007; Martin-Matthews, Tong, Rosenthal, & McDonald 2013). Chinese immigrants comprise the second largest population of immigrants in Canada (Statistics Canada, 2018) and, in Calgary, Chinese immigrants are the second largest visible minority group, constituting 6.0% of the population (Statistics Canada 2016). Despite older Chinese immigrants’ vulnerability, and cultural beliefs on death, which differ from Western populations, very limited attention has been paid to spousal bereavement among this population (Bennett et al., 2018; Martin-Matthews et al., 2013). Therefore, research into the lived experiences of spousal bereavement of this population is warranted.

## Literature Review

Numerous gerontology researchers have studied spousal bereavement and/or widowhood in terms of the effects of spousal loss and spousal bereavement adjustment (i.e., Donnelly & Hinterlong 2010; Halleröd 2013; Jin & Chrisatakis 2009; Johnson, Zhang, & Prigerson 2008; Tweedy & Guarnaccia 2008). However, very few studies have explored the lived experiences of spousal bereavement of aging populations via a qualitative approach, and further limited research is available which casts light on spousal bereavement experiences among older immigrants in general and from their own perspective, more specifically (Bennett et al., 2018; Martin-Matthews et al., 2013; Saito, 2013). This gap in scholarship impacts the provision of culturally-sensitive professional services with this population.

Three studies have examined the lived experiences of being widowed and/or spousal bereavement and among older immigrants residing in Western countries. Saito (2014) studied the bereavement experiences of nine Japanese immigrant widows aged 56 to 79, who had been in an international marriage in the United States. Saito found that cultural and religious factors supported women participants after the loss of their husbands, and widows reconnected with Japanese communities post-loss to acquire additional support. Martin-Matthews et al. (2013) conducted 20 in-depth interviews with Chinese widows aged 69 to 93 in Canada to reveal their widowhood experiences and access to social, financial and emotional support services. Study participants, who identified primarily as Chinese and as immigrants rather than as widows, reported receiving social supports from family and their ethno-cultural communities. A qualitative study by Bennett and colleagues (2018) found that loneliness and social isolation were central to bereavement adjustment among the eight Chinese older widows, aged 53 to 73, in their study. The researchers found that Chinese culture and beliefs assisted the widows in the study accepting the reality of spousal loss. We could not locate any studies specifically on the bereavement experiences of older immigrant men. Although this body of research is sparse, these three studies highlight the importance of cultural beliefs and connecting or reconnecting with their ethno-cultural communities in terms of coping with spousal loss and widowhood among older immigrant widows.

To address this knowledge deficit, we conducted a phenomenological study to understand the nature of spousal bereavement adjustment among older Chinese immigrant men and women living in Calgary, Canada. This study sought to answer the question: What are the lived experiences of widowed older Chinese immigrants in coping with their spousal bereavement?

## Methodology

We used a qualitative approach to explore the lived experiences of widowed older Chinese immigrants, which are multiple, varied and constructed by various social factors (Creswell, 2009). Descriptive phenomenology, as a theoretical lens, was employed as it allowed us to understand the meaning of a phenomenon (Davidsen, 2013), and to approach the phenomenon based on subjectivity of persons who have the experience by bracketing our own biases (Lopez & Willis, 2004).

This study received ethics approval from the Conjoint Faculties Research Ethics Board, University of Calgary and allparticipants provided written informed consent. Prior to beginning the interview participants were informed of the voluntary nature of their participation, including the right to refuse to answer any question, stop the interview, or withdraw from the study.

In-depth semi-structured qualitative interviews were conducted with 12 participants by two graduate social work students fluent in Mandarin or Cantonese who had clinical experience with older Chinese adults. Participants, who were first-generation Chinese immigrants living in Calgary for more than one year; aged 65 years of age or older; and were at least six-month post-loss of their spouse due to death, were recruited from faith communities, ethno-cultural communities, and service-providing organizations. The interviewing took place in 2018. Participants choose the locations for their interviews, such as their homes, senior living facilities, the nearby public libraries, churches, etc. The semi-structured interviewing guideline comprised, among other things, questions and subjects regarding the participants’ migration experiences, family relationships, marital loss realities, life after the spousal loss, and present daily routine.

A semi-structured interview guide was developed which included questions about their spousal loss and their immigration experiences, such as their relationship with their spouse, the death and funeral, family relationships, community activities, settlement and social networks in Calgary. Interviews were audiotaped and subsequently transcribed and translated into English, and verified for accuracy by the bilingual (Chinese and English) research team member. We employed Colaizzi’s (1978) method for data analysis in descriptive phenomenology as summarized by Wojnar and Swanson’s (2007) seven steps: (1) reading and rereading descriptions (interview transcriptions); (2) extracting significant statements of the phenomenon; (3) formulating meanings from significant statements to illustrate contexts of the phenomenon; (4) categorizing into theme clusters and validating with original data; (5) integrating the findings into description of the phenomenon; (6) returning to some participants to ask how it compared with their experiences; and (7) incorporating any changes offered by the participants (if available) into the final description of the essence of the phenomenon.

## Results

See Table 1 for the demographic profile of the participants. Study participants were nine female and three males ranging in age from 65 to 89, with an average age 76. All participants were widowed; one had remarried and shared her bereavement experiences in relation to her second husband. Eleven participants were retired; one was engaged in part-time work. In terms of living arrangements, four participants lived with their adult child, five lived alone in a senior apartment, two lived in their own house, and one resided in a rental room. Five identified as Christian, one as a Buddhist and the remaining six had no religious affiliation. Participants had lived in Calgary for approximately 14 years and experienced the death of their spouse approximately 11 years ago. Eight respondents in the study lost their spouse while living in their homeland, with spousal death serving as a major impetus to their immigration to Canada; four lost their spouse after migrating to Canada.

**Table 1:** Biographical information

No.	Name	Gender	Age	Language	Marital status	Residence	Employment	Years in Calgary	Years of spousal loss	Religious background
1	Lucy	Female	65	Mandarin	Widowed	Living in a rent room	Part-time job	9	14	Christian
2	Lan Hua	Female	65	Mandarin	Widowed	Living with son	Retired	9	25	Buddhism
3	Tom	Male	66	Cantonese	Widowed	Living alone in a senior apartment	Retired	22 (after 4 ys in Winnipeg)	4	/
4	Ying	Female	73	Mandarin	Widowed	Living with daughter’s family	Retired	7	15	/
5	Cui	Female	74	Cantonese	Widowed	Living alone in a senior apartment	Retired	19	3	Christian
6	Baoyu	Male	75	Mandarin	Widowed	Living in his own house with a renter	Retired	5	6	/
7	Song Jiaxiu	Female	78	Mandarin	Widowed	Living with daughter’s family	Retired	4	5	/
8	A Fang	Female	81	Mandarin	Widowed	Living with daughter’s family	Retired	8	7	Christian
9	San Gui	Female	82	Mandarin	Widowed	Living alone in a senior apartment	Retired	15	15	Christian
10	Jing	Male	84	Cantonese	Widowed	Living alone in his own house	Retired	24	0.5	Christian
11	Chow	Female	85	Cantonese	Widowed	Living alone in a senior apartment	Retired	20 (after 10 ys in Red Deer)	5	/
12	Liang	Female	89	Cantonese	Widowed	Living alone in a senior apartment	Retired	24	34	/

The following section presents the three main themes: long-lasting grief, spousal bereavement adjustment, immigration background, and each of their relevant subthemes. Quotations, drawn from the transcripts and labeled with the self-chosen pseudonym, gender and age of the participants, are used to illustrate each of the themes and subthemes.

### Long-Lasting Grief

Widowed older Chinese immigrants in the study reported experiencing long-lasting grief and bereavement, although they presented in different ways and to varying degrees. For instance, in referring to their loss, Ying (female, 73), said “his role could not be replaced by any other” and San Gui (female, 82) noted “my spouse was gone, and my root was gone.” Others expressed feelings of grief: “sadness”, “emptiness”, “loneliness”, in their depictions of spousal loss. The feelings associated with spousal loss are poignantly described in the following quotes:

When I am thinking of it, I am still sad…… Although he has gone, I don’t lead a comfortable life by myself. Why? I am thinking of him, thinking of him …… It’s very sad for me, only myself, to enter the home, thinking of him and crying for a while…... I think it is more horrible to the survivor than the deceased, because the survivor will think of the deceased all the time. (Ying, female, 73)

I feel very lonely. When she [my wife] was sick, [at least] we were together. It was totally different after she was gone. That feeling is particularly uncomfortable. I used to have such an idea that I don’t want to live any more. (Baoyu, male, 75)

Life after my wife passed away is almost the same. Not very different. I always cook for myself. But sometimes I just talk with the TV. Nobody is talking [with me]……I am kind of reluctant to part from her. I am still a little bit reluctant. After all we’ve been together for decades. (Jing, male, 82)

Some participants, particularly male respondents, noted their losses through alluding to the difficulties they experienced in meeting their instrumental needs, as 66-year-old Tom shared:

You can imagine that I was not alone at home before, but now I am at home by myself. Especially, I live here [a senior apartment], for example. You have to sign a contract every year. One of the questions [emergency contact] is whether you have family in case something would happen; someone could take care of me. I don’t know who I can write down.

### Spousal Bereavement Adjustment

**Fig. 1:**
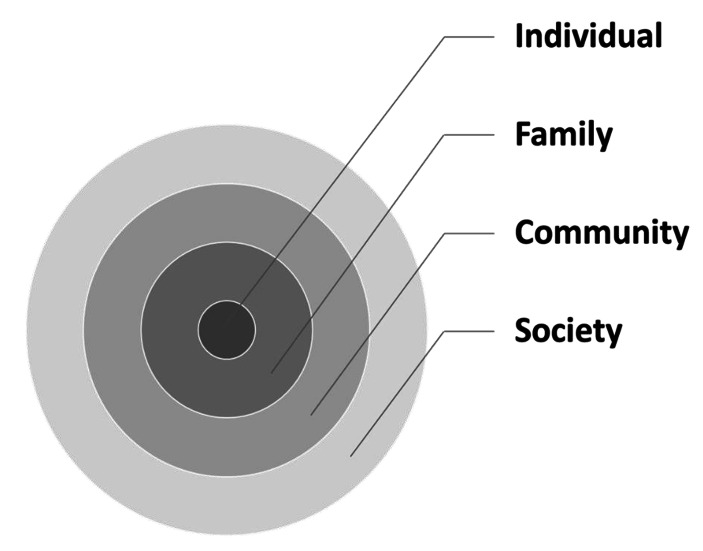
Lived experiences of spousal bereavement adjustment

Participants’ described their experiences of spousal bereavement adjustment according to individual, family, community, and societal levels (see Fig. 1).

#### Individual level

Bereavement occurring the individual level consists of personal cognitions, emotions and meanings attributed to spousal loss and personal action or behaviours related to grief. Within this domain participants chose to *keep their grief private*, mediate their grief through the performance of *cultural rituals*, and rely on their *faith or religious beliefs* to cope with their grief.

#### Keep grief private

Participants expressed their preferences for keeping grief and emotions related to their spousal loss private, relying on themselves to cope with spousal bereavement. They chose not to share their grief with others, especially with those who are close to them, as they “did not want to increase any pressure on the child’s shoulder” (Ying, female, 73). Sixty-five-year-old widow Lucy, shared that it was not necessary to talk about their grief since “no one could solve the problem”, as she explained:

Now I know I have to rely on only myself. No one else can solve your own personal experience. You have to rely on yourself. Slowly moving forward. .... I usually listen to songs. I don’t contact others much……Many people know me but never talk with me like what we [the conversation with the interviewer] are doing …… So, I told you, and you know that my life is very hard...… In fact, my inner emptiness has no way to fill up.

Similarly, A Fang (female, 81) commented, there is “no way to deal with loneliness” and in reference to being resigned to her loneliness, she stated: “Now I’m used to it anyway”.

#### Cultural rituals

Some participants used cultural rituals to maintain their bond with their deceased spouse. For example, San Gui (female, 82) has written a letter to her husband every year since he passed away 15 years ago. Song Jiaxiu (female, 78) explained her participation in the cultural ritual of burning ‘paper money’ to ensure the financial well-being of her deceased spouse: “last year I went back to China. I went to his cemetery to burn paper money to him. I went back the year before last year, too. It’s not easy to go back because of my age”.

Ying (female, 73) offered how viewing the image of her husband evoked sadness for her but was an important mechanism to preserve family memories:

I don’t dare to look at [his photo], because I am afraid [that I will feel too sad] by seeing him and thinking of him. I am thinking of taking our family photo next time I go back to China. I want to let my granddaughters see their grandfather.

#### Faith and religious beliefs

Faith and religious beliefs supported bereavement adjustment in helping participants accept the reality of spousal loss and manage their sorrow. For example, Lan Hua (female, 65) said she began to read books on Buddhism and started to visit the temple immediately after her husband died. She recalled resorting to her faith since she was unable to find someone to talk to about her bereavement:

After studying Buddhism, I learned that life is impermanent. Karma makes two people get together. When karma is gone, I have to face my life on my own……. I know the existence of people is not true. I view it [the spousal loss] with a more open mind……. What is dying is the “fake ego” and the “true ego” is eternal. When karma comes, a living body comes out. After karma is dispersed, the living body is free and returns to its original elements. Therefore, the “true ego” is eternal. The eternal thing, it still would show up by other objects. So, after you understand it, you don’t have to be too sad about it [the spousal loss].

Others believed there would be a reunion between them and their deceased partners once they die:

Since I believed in the Lord, and I believed Jesus. I said to God, now my husband has gone without the faith. Will you Lord arrange for me to be with my husband [when I die], because you are a kind, humble, loving shepherd. (San Gui, female, 82)

I am optimistic. My daughter’s child, she said I will see my wife in the heaven one day, I think so too. So, it doesn’t matter. I still have hope. The faith is the best…… I think we are Christians so there would be no problems. So, I’m not very sad when she’s gone. (Jing, male, 82)

Religious faith supported their bereavement for some older adults, as Cui noted:

It is good to go to the church, because the church is a big family, and the church makes me feel the sense of belonging. People there help each other. We also talk about the truth of life, because Jesus wants to save thousands of people. You don’t have to sink alone [in bereavement]. (female, 74)

#### Family level

Although participants acknowledged the *instrumental and emotional supports* provided by the family, they indicated that family was *unable to provide direct support* in relation to their grief.

#### Instrumental support

Participants in the study played an important role maintaining family life, which was often reciprocated through the instrumental support provided to older adults by their families, such as the provision of transportation to recreation, religious and cultural activities. Also, older immigrants sponsored by an adult child, were dependent on their child financially and for housing, etc., for 10 years, as the following portrays:

When I first came, it was the daughter who was committed to sponsor me for 10 years. My daughter took care of all of the problems…… You have to connect with your family doctor. I was not able to do that. I had to let my daughter make an appointment in advance…… When I first came, it was even hard for me to ask directions or go shopping…… I have no way to communicate with the doctor, I must have her [daughter]. (A Fang, female, 81)

The most difficult time was that when I first came here, I was sick. My son took me to the hospital and it was very troublesome…… Always my son helped me to buy rice and food. (Liang, female, 89)

Usually, I do not take a bus, and just stay at home. If I need to go out and do something my son or his friends will give me a ride. Otherwise, I would like to walk and take a bike. In winter, I don’t go out [on my own]; it is too cold. (Lan Hua, female, 65)

#### Psychological well-being

Family relationships were central in terms of participants’ emotional well-being. As A Fang (female, 81) described her relationship with her adult son:

Children are very filial. My eldest son supported me to paint, and the pens and papers he bought from China are very expensive…… My son and daughter-in-law like my paintings. They are all very filial. I don’t have to ask, but they would buy it for me. My son said, “Mom, don’t think about the price. Buy whatever you like. Wear whatever you like”.

Enjoyment from family relationships was particularly noted in their relationships with grandchildren, as 73 year old widow Ying explains:

She [youngest granddaughter] is sweet. If I am a little bit unhappy, she would say, “Granny, did I make you angry again?” I say, “No, I’m not angry”. She goes on to say, “I won’t make you angry”. Her mouth is so sweet. Before I go to bed, the older granddaughter would say, “Grandma, good night”. They are always like that.

#### Failure to offer direct support for older adults’ grief

As noted, family provided instrumental and psychological support for the widowed older adults in the study. However, since the widowed older adults hid their bereavement from family members, they were then unable to provide direct support for the spousal loss, as the following quotes illustrate:

I will not let her [my daughter] know. Because if I am sad, my daughter will be very sad as well. I will not let her know. I won’t say I was crying. Because she is sensitive. I don’t want her to worry about me, I don’t want my girl to feel stressful. (Ying, female, 73)

Sometimes, relatives came to comfort me [after the spousal death]. I felt more uncomfortable with that. Then I simply did not contact them. I went to the temple by myself, and listened to the morning classes and read books, and then I went to work. (Lan Hua, female, 65)

#### Community level

Participants in the study relied almost exclusively on Chinese-based social networks and community engagement for instrumental, social and emotional supports. Involvement in the Chinese *faith community* was promoted as important for both those with and without religious backgrounds. However, similar to family members, these community networks did not provide direct support for spousal bereavement according to participants in the study.

#### Faith community

For some respondents attending a faith community facilitated adjustment to spousal loss by allowing them to engage in community activities and social networks. In addition, faith communities provided instrumental assists, such as assisting the older adults in the study in dealing with family conflicts, applying for a senior apartment, and interpretation services. The following quotes illustrate the role of the faith community:

I go to the Buddhism school every Friday night. There are dozens of people. Everybody knows each other. Everybody is in the class together…… We contact each other with WeChat [a communication app], where we exchange information and communicate with each other. (Lan Hua, female, 65)

I go to church every week. I attend church activity every Sunday. I learn English there on Monday. There is a prayer meeting on Wednesday night. (A Fang, female, 81)

Now I am learning English in the church. There are many people every week, including those who are not Christians. Usually there are 50 or 60 people every time. First, we attend the English class from 9:00 to 11:00, then the tea meeting. Everyone is very happy, so there are a lot of people attending. I go there once a week. (A Fang, female, 81)

I haven’t believed in God yet, but my daughter does. I go to church every Sunday. I know some older women and older men there. (Song Jiaxiu, female, 78)

I go to the church every Monday and Saturday. I know a lot of friends here…… The church is my second home. (Jing, male, 82)

#### Societal level

The societal level refers to participants’ interactions within society, such as professional services from the public, private sectors and social sectors. In terms of bereavement experiences, most participants did not access services for bereavement support, and for those who had received services, they reported that the service were not appropriate.

#### Professional services for bereavement

Most respondents did not access services to specific coping with spousal bereavement as they were either not aware of the support or did not consider emotional health support necessary. Tom (male, 66) and Lucy (female, 65) were notable exceptions. Lucy emphasized her desire for functional supports related to housing and living costs, instead of services directed at facilitating coping with bereavement, as she stated:

There are groups for the widowed and for family relationships…… I was trying to get rid of my situation. It [the agency] played a certain role at that time. She [the counsellor] taught me to relax and breathe deeply. It played a certain role……The actual problem cannot be solved. Just chat, and then feel better. It is impossible for me to ask for a low rent housing. It is impossible to ask for living expenses.

Tom described receiving a letter of invitation from a government agency two months post spousal loss. He responded and received individual counselling, which he identified as a “failure” due to issues of cultural competence:

I’ve been there [counselling] four or five times. But I don’t think it’s good. I don’t think they understand our Chinese ideas…... They think differently from our Chinese people. He wants you to cry. “You cry whenever you want to”. But you know, we Chinese men don’t cry. But I don’t want to cry, how to cry...... They gave me a lot of paper materials [to manage my emotions]. Let me follow these to do something. I don’t think I need it.

### Immigration Background

Study participants were born in China/Hong Kong, with an average of 15 years since they had immigrated to Canada, and approximately 14 years of residence in Calgary. Their immigration background was implicated in their bereavement experiences.

#### Immigration for family reasons

In the Chinese context, cultural norms of filial piety imbue the responsibility of adult children to provide support to their aged parents and a strong expectation from Chinese older adults to receive family support (Cheng & Chan, 2006). Following these cultural norms, the adult child living in Canada is expected to be responsible for taking care of the surviving parent. One way to fulfill the obligations for filial piety is to support the parent to migrate to Canada following the loss of his or her spouse, as Ying (female, 73) identified:

I only have this one daughter. [When I was living in China] I thought I would have to live with her in the future. My siblings have their own families, right? I have to live with my daughter…… [If my husband didn’t die,] I won’t be here, I might just go back. Because he is not in good health, I have to stay with him. Right now, I don’t have him, if I go back [to China], my daughter in Canada would be worried [about me].

On reflecting on her decision to immigrate and the role of filial piety, A Fang (female, 81) noted:

I was getting older and I always had to think about some future events. What if my child is not around? After all, I have somebody [the daughter] to rely on. So that’s why I considered to come to my daughter here [in Canada].

Similarly, other participants noted:

He [my son] is a doctor and can give me a handy treatment. So I decided to come [to Canada]. ....... There is no one else in the family. He does not have any siblings. (Lan Hua, female, 65)

My two daughter-in-laws were deficient in filial piety and I was afraid that I could not live well even with their care. My daughter [in Canada] knew that her mother had suffered through a lifetime. She applied for a visitor visa for me first, for two years…… My daughter applied for my Permanent Residence in 2014. (Song Jiaxiu, female, 78)

#### Migratory grief and adjustment

The migratory histories of participants in the study had a powerful impact on their bereavement adjustment. Migratory grief, which is caused by a symbolic loss, such as loss of a homeland, status, social environment, and cultural identity (Casado et al., 2010) was foundational in most participants’ narratives. Participants attempted to maintain a strong bond with their homeland by following news and/or travelling back and forth between China/Hong Kong and Canada, post-migration. While in China they described cherishing the time visiting family or friends, and so on, as the following quotes illustrate:

Most of time I watched TV shows from Hong Kong. Usually I played the shows repeatedly……I try to have some connection with Hong Kong, and I try to know what is happening in Hong Kong. (Liang, female, 89)

I still would like to go back [to China] if I am in a good health. I want to go back when my grandson is getting married. (Song Jiaxiu, female, 78)

I went back to Hong Kong many times, more than 10. The last time I went back was last March to visit my mother. She is already 97 years old now. (Tom, male, 66)

#### Post-migration adaptation

Many participants shared their difficulties in adaptation post-migration. Adjusted to their host country in late life was noted as challenging, as the following quotes elucidate:

I didn’t get used to life here. I can’t understand what people are saying here. I think there are too few people here, no fun. (Ying, female, 73)

After I came, I was just like being in the jail at the beginning, so it was a lot to suffer. (San Gui, female, 82)

I didn’t get any additional help. However, some language problems are always bothering. (A Fang, female, 81)

[I didn’t get used to life in Canada] because of the traffic and social networking, and also because my belief [Buddhism] is different from theirs. (Lan Hua, female, 65)

## Discussion and Implications

Participants in this study exhibited long-lasting grief, which has been interpreted by some researchers as demonstrating respect for the deceased in Chinese culture (Prigerson et al., 2009). Older Chinese widowed immigrants strongly articulated their desire to grieve in private: they preferred to not share their emotions or memories related to spousal loss readily with family members or within their faith or ethno-cultural communities. They shared that this practice arose from the desire not to burden family members with their grief. Similarly, the concept of “ethic of emotional independence” coined by Martin-Matthews, Tong, Rosenthal, and McDonald (2013, pp. 513) illustrated the experience of grieving among a small sample (N = 20) of older Chinese widows living in Toronto. In a similar way the women in their study did not cite family as emotional support providers, instead they commented that their decision not to share their grief with family members was as a consequence to not wanting to burden family members. Additionally, a qualitative study that used three focus groups noted that death or dying in Chinese culture references bad luck, and consequently Chinese widows/widowers were less likely to discuss bereavement in the family or within their faith or ethno-cultural communities in order to avoid evoking bad luck (Yick & Gupta, 2002).

As a consequence of grieving in private, respondents relied primarily on their own coping processes to deal with their bereavement, adopting strategies such as maintaining a continuous bond with the deceased partner by practicing certain rituals, and/or using faith to accept the reality of the spousal loss and to manage their sorrow. In this study, the bereaved individual’s decision to maintain an ongoing bond was illustrated in participants’ continued reference to their deceased partner in relation to their role in the family (i.e., husband, wife, grandfather or grandmother). A model of continuing bond has been widely cited within the bereavement literature (e.g., Field 2006; Field, Gal-Oz, & Bonanno 2003; Harper, O’Connor, Dickson, & O’Carroll 2011; Packman, Horsley, Davies, & Kramer 2006). Continuing bonds refers to the ongoing inner connections with the deceased and inner representation of the deceased (Klass, Silverman, & Nickman 2014). Chinese scholars have likened traditional Chinese practices of bereavement to the continuing bonds model. For instance, in the annual Ching Ming Festival, Chinese people visit the graves of deceased family members and, in doing so, reaffirm their bond with the deceased (Jiang, 2005). Further research is necessary to promote a deeper conversation on the grieving practices of widowed older Chinese immigrants residing in Western settings and, specifically in relation to the appropriateness to the continuing bond model or other more culturally relevant grieving practices. Also an enriched understandings of the role of cultural in older Chinese immigrants coping with spousal loss is required to inform the development of culturally appropriate services and supports.

This study found that family provided instrumental supports for the older adults such as transportation and language interpretation, and that the psychological well-being of Chinese older adults in the study was frequently associated with positive familial intergenerational interaction. Studies conducted in China have underscored the importance of familial intergenerational interaction in terms of promoting older adults’ well-being. Tian (2016), for instance, identified that intergenerational support was positively associated with self-esteem and negatively associated with loneliness among Chinese older adults (N = 429). Further, receiving support from adult children was the key to Chinese older adults’ psychological well-being, regardless of the number of offspring or the gender of adult children (N = 3,039), according to Chen and Silverstein (2000).

Although participants in this study did not access bereavement support directly from their faith or ethno-cultural communities, they used these organizations for community engagement, overall psychological well-being and to acquire other instrumental supports. Two qualitative studies on older immigrant widows (Martin-Matthews et al., 2013; Saito 2014) came to similar conclusions, reinforcing the importance of faith and ethnic communities, wherein older immigrant widows and widowers can acquire multiple supports and social inclusion. Specifically,

In response to studies findings of the extremely limited reliance on family and faith and cultural community organization in providing direct support for spousal bereavement for Chinese immigrants in late life, consideration must be given regarding the development of culturally appropriate ways to involve families and these types of organizations in facilitating bereavement adjustment of older Chinese adults. For instance, programs related to grief and adjustment following spousal loss could be developed and implemented within faith and ethno-cultural communities. Also, attention should be paid to involving family members in these types of supports.

Only two participants in the study accessed professional bereavement support. Lack of knowledge about services and limited cultural sensitivity among service providers was noted by some participants as barriers to receiving professional mental health services. Similarly, considerable academic attention has been paid to health service barriers for older Chinese immigrants in North America. Service barriers among this population have been described as related to culture, language and ethnic difference between service providers and Chinese older immigrants (Dong et al., 2015; Lai & Chau, 2007). In addition, older Chinese immigrants reported feeling unwelcome and unwilling to access the care system even though they were in need of professional supports (Lai & Chau, 2007).

This study categorized participants’ experiences of spousal bereavement adjustment into individual, family, community, and societal levels (see Fig. 1). This approach is comparable to the ecological resilience framework put out by Windle and Bennett (2012). Their concept attempted to investigate how people interact with their living environments—individually, in communities, and in society, and to comprehend them in those situations, in the context of caring relationship. More research in the future should yield further insights, especially when examining older adults’ resilience or self-reliance capability and social support in the environment and how they would affect their lived experiences and/or quality of life.

Most study participants acknowledged having limited awareness of mental health services. In the same way, Sadavoy, Meier, and Ong (2004) found remarkably low levels of knowledge of professional mental health services among ethno-minority older adults, and specifically among Chinese older adults. Even among those in the study who reported being awareness of supportive services for bereavement, they expressed an unwillingness to access these supports. As an explanation, Lam et al. (2010) suggested that stigma associated with mental health issues might be rooted in Chinese religions and in societal collectivism. At the individual level stigma related to mental health concerns was associated with shame and guilt, which then acted to prevent this population from seeking support when faced with emotional issues. Further, many researchers have also identified the stigma and self-stigma of mental health issues in Chinese communities systematically prevented Chinese from professional mental health services (Kung, 2004; Sadavoy, Meier, & Ong 2004). These facts could have a negative impact on spousal bereavement adjustment among older Chinese immigrants, considering the reluctance to seek professional mental health services among this group. Culturally appropriate education programs aimed for removing stigma on mental health issues and enabling older immigrants with services information are required.

In the current study, many participants portrayed their grief in relation to their immigration experiences, expressing both physical and symbolic losses as a consequence of their move to Canada. Participants coped with migratory grief by following the news and events in their homeland, watching Chinese TV shows and through visits to their homeland. Migratory grief has been a subject of inquiry among immigrant groups and among older immigrants (Ahn, 2005; Casado & Leung, 2002; Lee, 2007; Lim et al., 2011). This grief must also be considered in the context of the wider loss of their extended family members, friends and communities as a consequence of migration. Especially, for those who immigrated to Canada after the spousal loss, migratory grief and spousal bereavement might exacerbate each other. Little research has been conducted to examine the relationship between migratory grief and spousal bereavement among immigrants in general. A 1999 qualitative study by Valencia-Go pointed out that spousal loss adjustment might be complicated by symbolic and physical losses of immigration among 14 older Filipino widows. Further research could explore the potentially dual impact of immigration and the spousal loss on older adults’ psychological well-being.

Because of their migratory experiences, Chinese older immigrants are vulnerable to mental health problems, such that scholars suggested the need for extra attention and services (Casado & Leung, 2002; Lai, 2004). This vulnerability might also contribute to complicated spousal bereavement experiences and greater challenges to grief adjustment. In addition to spousal loss and its associated grief, bereavement and adjustment, widowed older Chinese adults in the study were faced with the adjustment and grief related to migration. This places the older adults of this study in a double jeopardy: not only do they experience long-lasting grief and even complicated grief, the cultural factors including the ethic of emotional independence (Martin-Matthews et al., 2013) also impair their ability to access mental health supports. An intersectionality perspective should be utilized in working with older Chinese adults. Namely, practitioners should have adopt an anti-oppressive perspective to examine how ethnicity, immigration and age/ageing have influence on Chinese older immigrants’ well-being (Koehn et al., 2013). Practitioners should also be equipped with sufficient knowledge of the migratory background, immigration policy and related social welfares which affect older Chinese immigrants’ living situations, to understand their unique needs and the challenges they might face.

## Limitations

This study examined gender in a binary way, exclusively female and male. Those identifying as non-binary genders and those in same sex relationships or members of the LGBTQ populations were thus excluded. Many researchers have explored systematic challenges that LGBTQ and non-binary-identifying older adults were facing, such as social marginalization, discrimination from care sectors and so forth (Ansara, 2015; Schwinn & Dinkel, 2015; Sokolec & Dentato, 2014). Further research is supposed to engage older immigrant adults, including Chinese, from LGTBQ communities in terms of their spousal bereavement and widowhood. Also, although this study gathered the perspectives of older immigrants with ethnic minority backgrounds in one city in Canada, findings are not necessarily applicable to other ethnic communities, or other settings. Further studies should outreach other minority communities where older populations are facing similar challenging situations, such as social isolation and migratory stress. Finally, although some men were included in this sample, further research with immigrant men experiencing spousal lose is necessary to provide a more fulsome gendered account.

## Data Availability

The data that support the findings of this study are available on request from the corresponding author, WQ. The data are not publicly available due to ethical restrictions.
